# An Evaluation of Polish Women’s Knowledge of Perineal Incision: A Cross-Sectional Study

**DOI:** 10.3390/jcm14145142

**Published:** 2025-07-19

**Authors:** Romana Buchert, Katarzyna Wszołek, Kinga Bednarek, Marcin Wierzchowski, Maciej Wilczak, Karolina Chmaj-Wierzchowska

**Affiliations:** 1 Department of Maternal and Child Health and Minimally Invasive Surgery, Poznan University of Medical Sciences, 60-535 Poznan, Poland; romka.buchert@gmail.com (R.B.); kinga.bednarek@ump.edu.pl (K.B.); mwil@ump.edu.pl (M.W.); 2 Chair and Department of Chemical Technology of Drugs, Poznan University of Medical Sciences, 60-806 Poznan, Poland; mwierzch@ump.edu.pl

**Keywords:** perineal incision, episiotomy, knowledge of perineal incisions

## Abstract

**Background/Objectives**: A perineal incision (episiotomy) is a surgical procedure involving the controlled cutting of perineal tissues to widen the vaginal outlet during the second stage of labor. The aim of this study was to assess Polish women’s knowledge regarding perineal incision. **Methods**: This study was conducted using an unvalidated, anonymous questionnaire created in Google Forms. **Results**: Women with higher education, those who had undergone childbirth, and those who identified the Internet, medical personnel, medical personnel on social media, and medical journals as sources had significantly higher levels of knowledge. Respondents aged 25 years or younger had significantly lower knowledge levels compared to those aged over 26. Additionally, respondents living in cities with populations of up to 500,000 had significantly lower levels of knowledge compared to women living in larger cities. **Conclusions**: The level of women’s knowledge about perineal incision varies and is influenced by several factors. Significant determinants of higher levels of knowledge were higher education, having a history of obstetric delivery, being over 25 years old, and using information provided by medical personnel, including those present on social media.

## 1. Introduction

A perineal incision (episiotomy) is a surgical procedure involving the controlled cutting of perineal tissues, including the skin, vaginal mucosa, superficial transverse perineal muscle, and sometimes the anal levator muscle [1], to widen the vaginal outlet during the second stage of labor. In the modern approach—where evidence-based medicine (EBM) is implemented in clinical practice—it has been shown that the routine incision of the perineum is not beneficial and should be performed only according to strictly defined indications [2,3,4,5].

There are two types of perineal incision: median (midline) and mediolateral. When an incision is necessary, a mediolateral incision is recommended, as it is associated with a lower risk of grade III and IV perineal ruptures. In contrast, a midline incision clearly increases this risk [6,7]. Indications for episiotomy include the presence of life-threatening fetal symptoms (e.g., bradycardia requiring the faster termination of labor) [8], shoulder dystocia during labor [5], operative delivery (particularly with obstetric forceps) [9], fetal macrosomia, or other factors that increase the risk of injury to the mother and child [10]. The procedure is intended to reduce the risk of grade III and IV perineal ruptures [11,12]. According to World Health Organization (WHO) guidelines, episiotomy should be performed in no more than 10% of parturients [5]. However, Polish data indicate that episiotomies are still performed too frequently, with wide variation between hospitals—ranging from 20% to 86% of all births [13].

Evidence-based practice is a natural part of patient care—this approach promotes the implementation of management strategies supported by current data that are more likely to benefit than harm the patient [14,15,16,17,18,19]. However, the perineal incision procedure appears to deviate from this approach. The percentage of women who undergo episiotomy varies significantly depending on the country in which the data were collected [20,21]. Moreover, substantial discrepancies exist even within the same country, depending on the hospital [13,21]. An analysis of published studies on women’s awareness of perineal incision reveals significant differences among populations. Haji et al. [22] found that 30.3% of respondents in Saudi Arabia had never heard of episiotomy. In a Nigerian study, Inyang-Etoh et al. [23] reported that 32% of respondents were unfamiliar with the term before delivery. In another Nigerian study, nearly 19% of pregnant women surveyed had never heard of the procedure [24].

The aim of this study was to assess Polish women’s knowledge of perineal incision depending on age, education, the place of residence, past childbirth, and the source of knowledge, including the Internet, medical staff, medical staff on social media, and scientific articles.

## 2. Materials and Methods

Between 15 June 2023, and 30 July 2023, a total of 469 completed questionnaires were collected. This study was conducted using a self-created, unvalidated, anonymous questionnaire created in Google Forms (written in colloquial language understandable by all patients). Participation was voluntary and conducted entirely online. The inclusion criteria were female gender after natural childbirth. Patients with a history of cesarean section or vacuum forceps use, as well as male participants, were excluded.

The survey was distributed via the social media platforms Instagram and Facebook. The post containing the questionnaire was shared in the following groups:

1. “I give birth in Polna”—a support group for women giving birth in the Gynecological and Obstetrics Clinical Hospital named after H. Święcicki of the K. Marcinkowski Medical University in Poznań.

2. “I give birth in Raszei”—a support group for women giving birth in the Department of Gynecology and Obstetrics with Pregnancy Pathology, Franciszek Raszeja Municipal Hospital in Poznań.

3. “Studentawka”—a group that brings together both people directly interested in student issues and those who need support and help in this area. In addition, five people shared the questionnaire with their friends on Facebook.

The first section of the questionnaire collected basic demographic information, including age, education, the place of residence, sources of knowledge, and past natural childbirth (this was not subject to secondary verification). This study examined knowledge of various aspects of perineal incision: women’s knowledge of perineal incision, indications for perineal incision, and methods of perineal protection. We initially considered a composite knowledge score, but this approach was not retained following peer-review feedback, given the heterogeneity of knowledge items and lack of standard scoring criteria.

A statistical analysis was performed to evaluate Polish women’s knowledge of perineal incision depending on age, education, the place of residence, past childbirth, and the source of knowledge, including the *Internet*, medical staff, medical staff on *social media*, and scientific articles. Associations between categorical variables were tested using the Chi-square (χ^2^) test of independence.

Data analysis was performed using Statistica (Cloud Software Group, Inc., Fort Lauderdale, FL, USA (2023), *Data Science Workbench*, version 14) and Microsoft Excel (Microsoft Office, Redmond, WA, USA (2019), version 2205). A significance level of *p* < 0.05 was applied in all calculations.

## 3. Results

### 3.1. Group Characteristics

The age of respondents ranged from 18 to 65 years, with an average age of 28 years (29.09 ± 6.44). The characteristics of the study group—including age, education, the place of residence, declared connection with the medical community, childbirth experience, and sources of knowledge indicated by respondents—are presented in Table 1.

A natural childbirth was reported by 263 respondents (56.80%), among whom 186 (70.72%) experienced a perineal incision.

### 3.2. Women’s Knowledge of Perineal Incision

#### 3.2.1. What Is a Perineal Incision?

According to 432 respondents (93.30%), a perineal incision is a procedure; 11 respondents (2.38%) described it as surgery; and 20 (4.32%) reported having no knowledge of it. The results show that postpartum women and respondents who cited medical personnel as a source of knowledge were significantly more likely to know what a perineal incision is (Table 2).

#### 3.2.2. Is a Perineal Incision Performed Routinely?

Among respondents, 8 (1.73%) believed that a perineal incision is performed routinely, while 436 (94.17%) stated that it is not. Nineteen respondents (4.10%) reported having no knowledge of the subject. The results indicate that women with higher education, as well as those who identified sources such as medical personnel, social media, and medical personnel on social media, were significantly more likely to know that a perineal incision is not routinely performed on every woman (Table 3).

#### 3.2.3. Is the Consent of the Parturient Required to Perform a Perineal Incision?

According to 323 respondents (69.76%), the consent of the parturient is required to perform a perineal incision; 87 (18.79%) believed that consent is not required; and 53 respondents (11.45%) did not know. The results show that women over 30, those with higher education, those who had undergone childbirth, and those who identified the Internet, medical personnel, and medical personnel on social media as sources of knowledge were significantly more likely to know that the woman’s consent is required before performing a perineal incision (Table 4). Additionally, women who did not cite articles as a source of knowledge were significantly more likely to declare a lack of knowledge on this topic.

#### 3.2.4. Does the Parturient Have the Option to Refuse the Incision?

According to 345 respondents (74.51%), the parturient has the option to refuse a perineal incision; 24 respondents (5.18%) believed there is no such option; and 94 (20.305%) did not know. The results show that women over the age of 26, respondents who had undergone childbirth, and those who identified medical personnel and medical personnel on social media as sources were significantly more likely to know that the parturient has the right to refuse a perineal incision (Table 5).

#### 3.2.5. Is It Possible to Protect the Perineum from a Perineal Incision?

According to 391 respondents (84.45%), it is possible to protect the perineum from a perineal incision, 4 respondents (0.86%) believed there is no such possibility, and 68 (14.69%) did not know. The results show that women over the age of 26, respondents with higher education, those who had undergone childbirth, and those who identified the Internet, medical personnel, and medical personnel on social media as sources were significantly more likely to indicate that protection from a perineal incision is possible (Table 6).

#### 3.2.6. Does a Longer and More Painful Recovery Occur After Childbirth with a Perineal Incision Compared to a Wound After a Perineal Rupture?

According to 179 respondents (38.66%), a longer and more painful recovery is associated with a perineal incision during childbirth, 210 respondents (45.36%) indicated that a perineal rupture leads to a longer and more painful recovery, while 74 (15.98%) had no knowledge of the subject. The results show that women with higher education, women who did not undergo a perineal incision during childbirth, and respondents who identified medical personnel, social media, and medical personnel on social media as sources were significantly more likely to associate a longer and more painful recovery with a perineal incision (Table 7).

### 3.3. Knowledge of Indications for Perineal Incision and Methods of Perineal Protection

#### 3.3.1. Knowledge of Indications for Perineal Incision

In the questionnaire, knowledge of obstetric indications for performing a perineal incision during labor was assessed through a multiple-choice question. Respondents’ knowledge of these indications is presented in Table 8 and Table 9.

#### 3.3.2. Knowledge of Methods to Protect the Perineum

In the questionnaire, knowledge of methods to protect the perineum during childbirth was assessed using a multiple-choice question. Respondents’ knowledge of these methods is shown in Table 10.

The results show that women with higher education, women who had a connection with the medical community, women who had given birth, and women who indicated the Internet, medical personnel, medical personnel on social media, and medical journals as a source of knowledge were significantly more likely to indicate that such methods of protecting the perineum exist. The results indicate that women up to 25 years of age and those living in cities with fewer than 500 thousand inhabitants were more likely to indicate that the above methods of protecting the perineum do not exist.

## 4. Discussion

Perineal incision or perineal protection? Until recently, a surgical perineal incision to widen the birth canal and facilitate the course of labor was a routine procedure conducted during the second stage of natural childbirth. Currently, World Health Organization experts recommend the use of perineal incision in only 5% to 20% of births [5,25]. The proper preparation of perineal tissues, the option of delivering in a vertical position, and physical activity during pregnancy significantly increase the likelihood of childbirth without the need for an incision. In the present study, nearly 74% of respondents identified physical activity during pregnancy as a means of protecting the perineum, and 83% mentioned pelvic floor muscle exercises. Warm compresses were noted by nearly 42% of women.

Sun et al. [26] reported a significant reduction in the number of grade III and/or IV perineal injuries (RR 0.46, 95% CI 0.27–0.79), a decrease in the frequency of perineal incisions, and reduced perineal pain lasting up to 48 h postpartum in women giving birth for the first time. Similarly, Modoor et al. [27] observed a significant reduction in pain during the second stage of labor and a lower incidence of second- and third-degree perineal ruptures.

A Cochrane review by Aasheim et al. [28] confirmed that warm compresses reduce the risk of serious perineal injury. This is reflected in the guidelines issued by the Royal College of Midwives [29], which support the use of warm compresses. Only the hands-off technique has been associated with a reduction in the rate of perineal incisions, though without a corresponding impact on the severity of injury [28]. Therefore, the persistent belief among women—including those in the current study—that various techniques are effective for perineal protection remains puzzling. In a study by Trinh et al. [30], published in 2015, 76.8% of obstetricians and 82% of midwives stated that the primary purpose of a perineal incision is to prevent uncontrolled rupture. Additionally, 56.5% of obstetricians and 36.7% of midwives reported a lack of training in methods to minimize perineal injury. Similarly, in a 2021 publication, Yang et al. [31] found that 83.94% of obstetricians and 79.69% of midwives believed that performing a perineal incision had a protective effect against third- and fourth-degree perineal injuries. Furthermore, 80.29% of obstetricians and 82.57% of midwives identified a lack of training on perineal protection during childbirth as a major barrier to using alternative methods.

Carroll et al. [32] reported that Irish midwives expressed a strong need for further training and courses on perineal protection techniques and the management of perineal injuries. Among the respondents in the current study, women with a higher level of knowledge most frequently cited medical staff, medical professionals active on social media, and materials found online as sources of knowledge. In a study by Haji et al. [22], 49% of respondents reported the Internet and social media as their primary sources of information—these were the most frequently cited sources in that study group. In contrast, only 12.2% relied on information from medical professionals.

However, in a study conducted by Odo et al. [24], as many as 60% of the women in the study group indicated doctors and midwives as the people from whom they drew their knowledge related to the issue of perineal incision, and this was the main source of knowledge, while materials found on the Internet accounted for only 3.3%. It can be concluded that accessibility to sources of knowledge can vary significantly depending on the population.

The presence of medical personnel on social media is playing an increasingly important role in women’s health education, particularly on perinatal topics. For example, in this group, 93.9% of respondents were aware of the existence of perineal protection methods, and 80.8% indicated that a woman’s consent is required to perform an episiotomy. It can be assumed that the accessible format of content, the regularity of posts, and real-time interactions (e.g., Q&A sessions, live broadcasts) build trust in educators and increase the effectiveness of knowledge transmission. Similar observations were made by Ventola [33], who noted that social media can play a vital role in communicating medical information to patients and fostering engagement in the treatment process. Zhang et al. [34] emphasized that an increasing number of patients use social media as a source of health information. In a pilot study, Baker et al. [35] reported that 89% of pregnant women used social media to ask questions or seek advice related to pregnancy and/or parenting.

Marsh et al. [36] pointed out, however, that the presence of midwives on the analyzed social media platforms was minimal, which limits the visibility of nonmedicalized approaches to pregnancy and childbirth. This may be particularly important for promoting evidence-based knowledge, as information shared by medical professionals is often perceived as more reliable than content from other sources [34]. Medical staff on social media’ refers to healthcare professionals (e.g., midwives, obstetricians) sharing educational content via platforms like Instagram or Facebook. This is distinct from direct contact with providers in clinical settings.

In addition, Gholami-Kordkheili et al. [37] examined the growing online presence of healthcare professionals and its implications for public health, emphasizing the importance of ethical standards, professionalism, and evidence-based content. This phenomenon reflects a broader shift toward digital medicine (e-health), where the educational role of medical personnel extends beyond clinics, hospitals, and birthing schools. The responsible digital engagement of doctors, midwives, and other healthcare professionals promotes greater health literacy and may contribute to more informed participation by women in the birthing process.

In our study, age, education, and childbirth history were significant predictors of knowledge, depending on the area analyzed. Women over 30 and those with childbirth experience demonstrated significantly higher knowledge levels regarding episiotomy. The age group under 25 had the least knowledge, highlighting the need to intensify educational efforts targeted at younger women, particularly those who have not yet given birth. Similar findings have been reported by other authors, who observed higher knowledge levels among women with previous childbirth experience [22,38,39,40].

Failure to inform the patient or performing the procedure without consent or against her will can be considered a form of institutional violence and may constitute obstetric violence. Obstetric violence refers to actions and omissions by medical personnel that violate a woman’s bodily and psychological integrity during pregnancy, during labor, and postpartum—often justified by “the welfare of the child” or “medical necessity” [41]. In qualitative studies, women described being unable to refuse incisions, receiving no explanation for the intervention, and experiencing a lack of control over their bodies—factors contributing to traumatic childbirth memories [41,42]. Recognizing the right to informed consent and refusal is not only a legal aspect but also a key measure in preventing obstetric violence. It helps foster a sense of empowerment and safety for the parturient. In our study, a self-developed questionnaire written in colloquial language understandable by all patients was administered electronically and distributed through social networks and support groups. Online studies (e.g., Internet surveys) are often created by older, less educated, and rural people and may not be representative of these groups. There is also no way to verify a natural birth history.

Validated tools are commonly used in research; alternatively, “global assessment” measures based on direct self-reporting [43,44] or online surveys [45] are also used. While validated instruments offer greater objectivity, they are often time-consuming. Non-validated instruments, such as “global assessments”, are easier to administer but may be more subjective [46].

The results indicate a clear need to intensify educational efforts regarding the perineal incision procedure—its indications, alternatives, and potential consequences. Particular attention should be directed toward educating younger women who have not yet given birth, as this group reported the lowest level of knowledge. Social media can serve as an effective tool in this process, provided that content is created by qualified professionals and grounded in current guidelines and evidence-based medicine. It is also essential to emphasize every woman’s right to give or withhold consent for any medical intervention during childbirth. The findings of this study may serve as a foundation for future educational and organizational initiatives aimed at increasing women’s awareness and improving the standards of perinatal care in Poland.

## 5. Conclusions

The level of women’s knowledge about perineal incision varies and is influenced by several factors. Significant determinants of higher levels of knowledge were higher education, having a history of obstetric delivery, being over 25 years old, and using information provided by medical personnel, including those on social media.

## Figures and Tables

**Table 1:** Characteristics of the study group.

		*n* = 463 (%)
Age	<25 years	135 (29.16%)
	26–29 years	186 (40.17%)
	>30 years	142 (30.67%)
Education	Primary	1 (0.22%)
	Vocational	12 (2.59%)
	Secondary	106 (22.89%)
	Higher	344 (74.30%)
Place of residence	Rural	139 (30.02%)
	City < 100 thousand	65 (14.04%)
	City 100–500 thousand	47 (10.15%)
	City > 500 thousand	212 (45.79%)
Sources of knowledge	Medical journals	43 (9.29%)
	Medical books	77 (16.63%)
	Scientific articles	86 (18.57%)
	Internet	89 (19.22%)
	Friends	124 (26.78%)
	Social media	148 (31.97%)
	Medical staff on social media	198 (42.76%)
	Medical staff	293 (63.28%)
	Internet	311 (67.17%)
Past natural childbirth	Yes	263 (56.80%)
	No	200 (43.20%)

**Table 2:** Knowledge of perineal incision.

		Perineal Incision			*χ^2^*	*p*
		Procedure	Surgery	Do Not Know		
Has undergone childbirth	Yes	252 (95.82%)	3 (1.14%)	8 (3.04%)	*6.62*	*0.04*
	No	180 (90%)	8 (4%)	12 (6%)		
Source of knowledge was medical staff	Yes	282 (96.25%)	5 (1.71%)	6 (2.05%)	*11.3*	*0.004*
	No	150 (88.24%)	6 (3.53%)	14 (8.24%)		

**Table 3:** Knowledge of whether a perineal incision is a routine procedure.

		Routine Performance of Perineal Incision in Every Woman			*χ^2^*	*p*
		Yes	No	Do Not Know		
Education	Higher	4 (1.16%)	332 (96.51%)	8 (2.33%)	*11.73*	*0.003*
	Other	4 (3.36%)	104 (87.39%)	11 (9.24%)		
Source of knowledge was medical staff	Yes	4 (1.37%)	285 (97.27%)	4 (1.37%)	*15.57*	*<0.001*
	No	4 (2.35%)	151 (88.82%)	15 (8.82%)		
Source of knowledge was *social media*	Yes	4 (2.7%)	143 (96.62%)	1 (0.68%)	*9.56*	*0.01*
	No	4 (1.27%)	293 (93.02%)	18 (5.71%)		
Source of knowledge was medical staff on *social media*	Yes	4 (2.02%)	193 (97.47%)	1 (0.51%)	*14.52*	*0.001*
	No	4 (1.51%)	243 (91.7%)	18 (6.79%)		

**Table 4:** Knowledge of the need to obtain consent from the parturient for a perineal incision.

		Consent of Parturient to Make Perineal Incision			*χ^2^*	*p*
		Yes	No	Do Not Know		
Age	<25 years	83 (61.48%)	28 (20.74%)	24 (17.78%)	*21.37*	*<0.001*
	26–29 years	102 (69.39%)	23 (15.65%)	22 (14.97%)		
	>30 years	138 (76.24%)	36 (19.89%)	7 (3.87%)		
Education	Higher	253 (73.55%)	57 (16.57%)	34 (9.88%)	*8.81*	*0.01*
	Other	70 (58.82%)	30 (25.21%)	19 (15.97%)		
Past childbirth	Yes	207 (78.71%)	47 (17.87%)	9 (3.42%)	*43.13*	*<0.001*
	No	116 (58%)	40 (20%)	44 (22%)		
Source of knowledge was Internet	Yes	229 (73.63%)	51 (16.4%)	31 (9.97%)	***6.6***	***0.04***
	No	94 (61.84%)	36 (23.68%)	22 (14.47%)		
Source of knowledge was medical staff	Yes	232 (79.18%)	45 (15.36%)	16 (5.46%)	***39.26***	***<0.001***
	No	91 (53.53%)	42 (24.71%)	37 (21.76%)		
Source of knowledge was medical staff on *social media*	Yes	160 (80.81%)	27 (13.64%)	11 (5.56%)	***22.48***	***<0.001***
	No	163 (61.51%)	60 (22.64%)	42 (15.85%)		
Source of knowledge was scientific articles	Yes	31 (72.09%)	11 (25.58%)	1 (2.33%)	***6.06***	***0.048***
	No	292 (69.52%)	76 (18.1%)	52 (12.38%)		

**Table 5:** Knowledge of whether the parturient can refuse a perineal incision.

		Possibility for the Parturient to Refuse the Incision			*χ^2^*	*p*
		Yes	No	Do Not Know		
Age	<25 years	89 (65.93%)	6 (4.44%)	40 (29.63%)	*9.95*	*0.04*
	26–29 years	114 (77.55%)	9 (6.12%)	24 (16.33%)		
	>30 years	142 (78.45%)	9 (4.97%)	30 (16.57%)		
Past childbirth	Yes	216 (82.13%)	13 (4.94%)	34 (12.93%)	*21.03*	*<0.001*
	No	129 (64.5%)	11 (5.5%)	60 (30%)		
Source of knowledge was medical staff	Yes	243 (82.94%)	14 (4.78%)	36 (12.29%)	*32.14*	*<0.001*
	No	102 (60%)	10 (5.88%)	58 (34.12%)		
Source of knowledge was medical staff on *social media*	Yes	163 (82.32%)	10 (5.05%)	25 (12.63%)	*13.41*	*0.001*
	No	182 (68.68%)	14 (5.28%)	69 (26.04%)		

**Table 6:** Knowledge of the possibility to protect the perineum from incision during childbirth.

		Possibility to Protect the Perineum from Incision			*χ^2^*	*p*
		Yes	No	Do Not Know		
Age	<25 years	95 (70.37%)	1 (0.74%)	39 (28.89%)	*35.66*	*<0.001*
	26–29 years	126 (85.71%)	1 (0.68%)	20 (13.61%)		
	>30 years	170 (93.92%)	2 (1.1%)	9 (4.97%)		
Education	Higher	303 (88.08%)	0 (0%)	41 (11.92%)	*19.38*	*<0.001*
	Other	88 (73.95%)	4 (3.36%)	27 (22.69%)		
Past childbirth	Yes	246 (93.54%)	2 (0.76%)	15 (5.7%)	*40.3*	*<0.001*
	No	145 (72.5%)	2 (1%)	53 (26.5%)		
Source of knowledge was Internet	Yes	278 (89.39%)	2 (0.64%)	31 (9.97%)	*16.66*	*<0.001*
	No	113 (74.34%)	2 (1.32%)	37 (24.34%)		
Source of knowledge was medical staff	Yes	273 (93.17%)	2 (0.68%)	18 (6.14%)	*45.76*	*<0.001*
	No	118 (69.41%)	2 (1.18%)	50 (29.41%)		
Source of knowledge was medical staff on *social media*	Yes	186 (93.94%)	0 (0%)	12 (6.06%)	*27.63*	*<0.001*
	No	205 (77.36%)	4 (1.51%)	56 (21.13%)		

**Table 7:** Knowledge of recovery from perineal incision or rupture.

		Longer and More Painful Recovery			*χ^2^*	*p*
		Perineal Incision	Perineal Rupture	Do Not Know		
Education	Higher	143 (41.57%)	145 (42.15%)	56 (16.28%)	*6.07*	*0.048*
	Other	36 (30.25%)	65 (54.62%)	18 (15.13%)		
Childbirth without a perineal incision	Yes	68 (36.56%)	82 (44.09%)	36 (19.35%)	*8.89*	*0.01*
	No	43 (55.84%)	26 (33.77%)	8 (10.39%)		
Source of knowledge was medical staff	Yes	144 (49.15%)	112 (38.23%)	37 (12.63%)	*39.11*	*<0.001*
	No	35 (20.59%)	98 (57.65%)	37 (21.76%)		
Source of knowledge was *social media*	Yes	73 (49.32%)	58 (39.19%)	17 (11.49%)	*10.93*	*0.004*
	No	106 (33.65%)	152 (48.25%)	57 (18.1%)		
Source of knowledge was medical staff on *social media*	Yes	111 (56.06%)	63 (31.82%)	24 (12.12%)	*44.59*	*<0.001*
	No	68 (25.66%)	147 (55.47%)	50 (18.87%)		

**Table 8:** Knowledge of indications for perineal incision.

	*n* (%)
Acute fetal distress in final stage of labor	313 (67.60%)
Symptoms of threatened perineal rupture	262 (56.59%)
Use of obstetric forceps or vacuum extractor during labor	226 (48.81%)
Delivery of large fetus	219 (47.30%)
Oblique positioning of fetus head	98 (21.17%)
Faster completion of second period of labor	98 (21.17%)
Manual assistance in pelvic position of fetus	86 (18.57%)
Do not know	44 (9.50%)

**Table 9:** Knowledge of indications for perineal incision depending on age and education, place of residence, childbirth without a perineal incision, source of knowledge.

Age	<25 Years	26–29 Years	>30 Years	*χ^2^*	*p*
Use of obstetric forceps or vacuum extractor during labor	41 (30.37%)	79 (53.74%)	106 (58.56%)	*27.28*	*<0.001*
Acute fetal distress in final stage of labor	67 (49.63%)	110 (74.83%)	136 (75.14%)	*27.19*	*<0.001*
Faster completion of second period of labor	16 (11.85%)	31 (21.09%)	51 (28.18%)	*12.99*	*0.002*
Manual assistance in pelvic position of fetus	13 (9.63%)	35 (23.81%)	38 (20.99%)	*11.53*	*0.003*
**Education**		**Higher**	**Other**		
Use of obstetric forceps or vacuum extractor during labor		183 (53.2%)	43 (36.13%)	*10.42*	*0.001*
Acute fetal distress in final stage of labor		250 (72.67%)	63 (52.94%)	*15.17*	*<0.001*
Delivery of large fetus		151 (43.9%)	68 (57.14%)	*6.23*	*0.01*
**Place of residence**	**Rural**	**City** **<500.000**	**City** **>500.000**		
Use of obstetric forceps or vacuum extractor during labor	67 (48.2%)	44 (39.29%)	115 (54.25%)	*6.63*	*0.04*
Manual assistance in pelvic position of fetus	18 (12.95%)	19 (16.96%)	49 (23.11%)	*6.1*	*0.047*
**Childbirth without a perineal incision**		**Yes**	**No**		
Use of obstetric forceps or vacuum extractor during labor		150 (57.03%)	76 (38%)	*16.59*	*<0.001*
Acute fetal distress in final stage of labor		194 (73.76%)	119 (59.5%)	*10.51*	*0.001*
Faster completion of second period of labor		70 (26.62%)	28 (14%)	*11.21*	*0.001*
Do not know		9 (3.42%)	35 (17.5%)	*26.86*	*<0.001*
**Source of knowledge was Internet**		**Yes**	**No**		
Use of obstetric forceps or vacuum extractor during labor		162 (52.09%)	64 (42.11%)	*4.09*	*0.04*
Acute fetal distress in final stage of labor		228 (73.31%)	85 (55.92%)	*13.8*	*<0.001*
Do not know		22 (7.07%)	22 (14.47%)	*6.15*	*0.01*
**Source of knowledge was medical staff**		**Yes**	**No**		
Use of obstetric forceps or vacuum extractor during labor		158 (53.92%)	68 (40%)	*8.39*	*0.004*
Acute fetal distress in final stage of labor		223 (76.11%)	90 (52.94%)	*25.95*	*<0.001*
Manual assistance in pelvic position of the fetus		66 (22.53%)	20 (11.76%)	*8.7*	*0.003*
Do not know		13 (4.44%)	31 (18.24%)	*22.9*	*<0.001*
**Source of knowledge was *social media***		**Yes**	**No**		
Use of obstetric forceps or vacuum extractor during labor		60 (40.54%)	166 (52.7%)	*5.99*	*0.01*
**Source of knowledge was medical staff on *social media***		**Yes**	**No**		
Use of obstetric forceps or vacuum extractor during labor		114 (57.58%)	112 (42.26%)	*10.67*	*0.001*
Acute fetal distress in final stage of labor		163 (82.32%)	150 (56.6%)	*35.77*	*<0.001*
Symptoms of threatened perineal rupture		98 (49.49%)	164 (61.89%)	*7.08*	*0.01*
Delivery of large fetus		80 (40.4%)	139 (52.45%)	*6.63*	*0.01*
Manual assistance in pelvic position of fetus		45 (22.73%)	41 (15.47%)	*3.91*	*0.048*
Do not know		9 (4.55%)	35 (13.21%)	*10.7*	*0.001*
**Source of knowledge was scientific articles**		**Yes**	**No**		
Use of obstetric forceps or vacuum extractor during labor		29 (67.44%)	197 (46.9%)	*6.69*	*0.01*
Acute fetal distress in final stage of labor		38 (88.37%)	275 (65.48%)	*10.97*	*0.001*
Oblique positioning of fetus head		18 (41.86%)	80 (19.05%)	*10.48*	*0.001*
Manual assistance in pelvic position of fetus		21 (48.84%)	65 (15.48%)	*22.95*	*<0.001*
Do not know		0 (0%)	44 (10.48%)	*9.04*	*0.003*

**Table 10:** Methods of perineal protection according to respondents.

	*n* (%)
Perineal massage	397 (85.75%)
Pelvic floor muscle exercises	385 (83.15%)
Physical activity during pregnancy	341 (73.65%)
Childbirth in the knee and elbow position	225 (48.60%)
Childbirth in the crouching position	216 (46.65%)
Obstetric gels	195 (42.12%)
Warm compress	193 (41.68%)
Childbirth in the standing position	172 (37.15%)
None	11 (2.38%)

## Data Availability

The data presented in this study are available upon request from the corresponding author.
